# Have attacks on healthcare become the new normal? a public health call to action for armed conflicts before it is too late

**DOI:** 10.1186/s13031-023-00555-4

**Published:** 2023-12-06

**Authors:** Karl Blanchet, Leonard Rubenstein, Bertrand Taithe, Larissa Fast

**Affiliations:** 1 Geneva Centre of Humanitarian Studies, Faculty of Medicine, University of Geneva, Genève, Switzerland; 2 Johns Hopkins Bloomberg School of Public Health, Baltimore, USA; 3 Humanitarian and Conflict Response Institute, University of Manchester, Manchester, England

**Keywords:** Attacks, Threat, Violence, War, Conflict, International humanitarian law, Health professionals, Hospitals, Ambulance

## Abstract

The scale of attacks on healthcare has become more visible and its impact greater in recent armed conflicts in Ukraine, Sudan and Myanmar. In these conflicts, combatants systematically target health facilities and ambulances. We need to ensure that attacks on healthcare do not become the new norm amongst governmental troops and non-State armed groups. There is limited evidence about why and how attacks on healthcare have become “normal” practice amongst many combatants, despite the likely tactical and strategic costs to themselves. We are convinced that the problem now needs to be tackled like any other public health issue by assessing: the scale of the problem; who is the most at risk; identifying risk factors; developing new interventions to prevent the risks or address the issue; and evaluating the effectiveness of these interventions.

Attacks on healthcare have taken various shapes and have become a major concern in many countries and contexts. Health personnel have been aggressed by patients, family members and armed forces to the point that it is estimated that in the world about every second one health professional is a victim of violence at their workplace [1]. The situation has worsened during the COVID-19 pandemic [2]. Colleagues have launched a call to individual and collective action to prevent violence and protect healthcare workers [3]. The safety and protection for healthcare workers in contexts of armed conflicts, however, is even more precarious. Recent armed conflict in Syria, Myanmar, Ukraine and Sudan have escalated the destruction of health facilities and ambulances and the direct violence and threats exerted directly on healthcare workers.

The problem has worsened in the last few years, with belligerents willfully violating the laws of war as defined by the Geneva Conventions [4]. The Conventions explicitly prohibit “attacking the wounded or the sick and assaulting or punishing military or civilian health workers who offer them care, or inflicting violence on hospitals or ambulances” [5].

Significant efforts have been invested over the years to document the scale of problem: the WHO set up the surveillance system for attacks on health care (SSA); the Safeguarding Health in Conflict Coalition (SHHC), composed of around 30 member organisations, has published its annual state of attacks on healthcare in armed conflict since 2014, reflecting on the state of the issue; and the NGO Insecurity Insight has been collecting, analysing and publishing data from various sources while also promoting open access. The issue has gained in importance in the press and scientific journals [6] thanks to the work of strong champions advocating for the protection of health professionals and hospitals and against impunity for perpetrators [5, 7, 8].

However, it seems that the scale of the problem has become more visible and its impact greater in recent armed conflicts in Ukraine, Sudan and Myanmar. In these conflicts, combatants systematically target health facilities and ambulances despite all the efforts invested by the International Committee of the Red Cross and other humanitarian agencies to train and increase the awareness of soldiers on the International Humanitarian Law. If counting and documenting the incidence of attacks is not good enough as a deterrent for belligerents to change their ways, perhaps other evidence could be useful to change how these issues are debated. Documenting the impact of attacks on populations and health systems and proving that targeting health infrastructures and personnel will prevent combatants from gaining the heart of populations might carry as much weight as an appeal to the law. This is why we, a group of researchers, have joined efforts through the Researching the Impact of Attacks on Healthcare (RIAH) [9] project to develop new methods and generate new evidence. There is now more and stronger evidence of the impact of attacks on health outcomes, changes to health-seeking behaviour of patients who feel unsafe in hospitals, the morale of healthcare workers who leave their post because they fear for their life, the disruption of essential healthcare services and closure of facilities. By extrapolation, these point to significant and long-term costs for the functioning and reconstruction of health systems [6, 10]–[14].

The next step is to ensure that attacks on healthcare do not become the new norm amongst dictators and amongst their troops. There is limited evidence about why and how attacks on healthcare have become “normal” practice amongst many combatants, despite the likely tactical and strategic costs to themselves. However, there is an important challenge in front of us [15]: how do we challenge or change an implicit norm of behaviour [13, 16]? The challenge is even more vexing when considering cross-cultural variations or individual emotions and perceptions of risk. The fear of sanctions can constitute an incentive for behavioural change, [17] as other factors can be linked to social learning and trust in the person who recommends new norms [18].

To influence the dynamics of social norms on attacks on healthcare amongst leaders and combatants, we suggest adopting a new strategy by declaring attacks on healthcare as a global public health issue. We acknowledge that attacks on healthcare, like any other form of violence, are detrimental to human life and morale, and have long-term impact on health systems already affected by armed conflict and, by extension, inflict a huge societal cost to countries. We are convinced that the problem now needs to be tackled like any other public health issue by assessing: the scale of the problem; who is the most at risk; identifying risk factors; developing new interventions to prevent the risks or address the issue; and evaluating the effectiveness of these interventions [3].

In a similar approach to the one recommended by Kuhlmann et al. [16] regarding general violence against healthcare workers, we formulate a public health call to action with the following recommendations as a start:

- Train healthcare workers in conflict-affected countries on the risks they may experience,
- Identify appropriate prevention and protection measures for healthcare workers (particularly local healthcare workers) and their patients, and coping mechanisms to deal with mental health-related issues;
- Protect healthcare workers. This requires influencing national and global policies related to attacks on healthcare in humanitarian settings by using scientific evidence; developing guidelines for all combatants and training them on international norms; providing evidence and employing accountability mechanisms when the Geneva Conventions are not respected; offering managerial and mental health support to healthcare workers, particularly those working in national or local facilities, who are victims of threats and attacks;
- Establish independent reporting systems of attacks and their impact; multiply research programmes including implementation research;
- Engage the general public, media and communities, including armed forces, about the scale and impact of the problem, with scientists collaborating with human rights activists in a global campaign and national campaigns.

## Data Availability

NA.
