# Correction: Have attacks on healthcare become the new normal? A public health call to action for armed conflicts before it is too late

**DOI:** 10.1186/s13031-023-00564-3

**Published:** 2024-01-30

**Authors:** Karl Blanchet, Leonard Rubenstein, Bertrand Taithe, Larissa Fast

**Affiliations:** 1 Geneva Centre of Humanitarian Studies, Faculty of Medicine, University of Geneva, Genève, Switzerland; 2 Johns Hopkins Bloomberg School of Public Health, Baltimore, USA; 3 Humanitarian and Conflict Response Institute, University of Manchester, Manchester, England

**Correction: Blanchet et al. Conflict and Health _#####################_**

10.1186/s13031-023-00555-4

In this article the author name Rubenstein was incorrectly written as Rubinstein.

The original article (1) has been revised.

